# Acute cigarette smoke‐induced eQTL affects formyl peptide receptor expression and lung function

**DOI:** 10.1111/resp.13960

**Published:** 2020-10-19

**Authors:** Simon D. Pouwels, Valerie R. Wiersma, Immeke E. Fokkema, Marijn Berg, Nick H.T. ten Hacken, Maarten van den Berge, Irene Heijink, Alen Faiz

**Affiliations:** ^1^ Department of Pathology and Medical Biology University Medical Center Groningen (UMCG), University of Groningen Groningen The Netherlands; ^2^ Department of Pulmonology University Medical Center Groningen (UMCG), University of Groningen Groningen The Netherlands; ^3^ Groningen Research Institute for Asthma and COPD (GRIAC), University Medical Center Groningen (UMCG), University of Groningen Groningen The Netherlands; ^4^ Department of Hematology Cancer Research Center Groningen, University Medical Center Groningen (UMCG), University of Groningen Groningen The Netherlands; ^5^ Respiratory Bioinformatics and Molecular Biology University of Technology Sydney Sydney NSW Australia

**Keywords:** chronic obstructive pulmonary disease, cigarette smoking, formyl peptide receptor, gene expression, quantitative trait loci

## Abstract

**Background and objective:**

Cigarette smoking is one of the most prevalent causes of preventable deaths worldwide, leading to chronic diseases, including chronic obstructive pulmonary disease (COPD). Cigarette smoke is known to induce significant transcriptional modifications throughout the respiratory tract. However, it is largely unknown how genetic profiles influence the smoking‐related transcriptional changes and how changes in gene expression translate into altered alveolar epithelial repair responses.

**Methods:**

We performed a candidate‐based acute cigarette smoke‐induced eQTL study, investigating the association between SNP and differential gene expression of FPR family members in bronchial epithelial cells isolated 24 h after smoking and after 48 h without smoking. The effects FPR1 on lung epithelial integrity and repair upon damage in the presence and absence of cigarette smoke were studied in CRISPR‐Cas9‐generated lung epithelial knockout cells.

**Results:**

One significant (FDR < 0.05) inducible eQTL (rs3212855) was identified that induced a >2‐fold change in gene expression. The minor allele of rs3212855 was associated with significantly higher gene expression of FPR1, FPR2 and FPR3 upon smoking. Importantly, the minor allele of rs3212855 was also associated with lower lung function. Alveolar epithelial FPR1 knockout cells were protected against CSE‐induced reduction in repair capacity upon wounding.

**Conclusion:**

We identified a novel smoking‐related inducible eQTL that is associated with a smoke‐induced increase in the expression of FPR1, FPR2 and FPR3, and with lowered lung function. in vitro FPR1 down‐regulation protects against smoke‐induced reduction in lung epithelial repair.

## Graphical abstract

A candidate‐based inducible eQTL study was performed in occasional smokers, identifying rs3212855 flanking the FPR as novel cigarette smoke‐induced eQTL. In addition, rs3212855 was associated with lower lung function. Lastly, CRISPR‐Cas9‐downregulated FPR1 alveolar epithelial cells were protected against the harmful effects of cigarette smoke on epithelial repair.

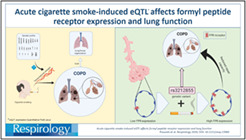

**Abbreviations**
ANOVA: analysis of variance
CRP: C‐reactive protein
CSE: cigarette smoke extract
DAMP: damage‐associated molecular pattern
ECIS: Electric Cell‐substrate Impedance Sensing
eQTL: expression quantitative trait loci
FEV_1_: forced expiratory volume in 1 s
fMLP: N‐Formylmethionine‐leucyl‐phenylalanine
FPR: formyl peptide receptor
FVC: forced vital capacity
gRNA: guide RNA
KLK1: Kalikrein 1
KO: knockout
RV: residual volume
SNP: single nucleotide polymorphism

## INTRODUCTION

According to the World Health Organization, over 1.1 billion individuals smoke cigarettes on a regular basis, with 36.1% of all males and 6.8% of all females worldwide being smokers.^1^ Unsurprisingly, considering the well‐known adverse health effects, cigarette smoking remains one of the leading causes of preventable morbidity and mortality to date. It may lead to various diseases, including chronic obstructive pulmonary disease (COPD), cardiovascular diseases and cancer. COPD is a lung disease characterized by chronic inflammation, leading to chronic airway obstruction (chronic bronchitis) and loss of alveolar structures (emphysema). Not all chronic smokers develop COPD, being caused by the combination of chronic environmental exposures, with cigarette smoke being the main risk factor, and genetic susceptibility. Cigarette smoke is a complex mixture consisting of more than 5000 chemicals, many of which are toxic or carcinogenic. For 98 of these chemicals, it has been shown that the toxic threshold is exceeded upon inhaling mainstream cigarette smoke.^2^ The respiratory epithelial layer is the first line of defence upon inhalation of cigarette smoke, which acts as a physical and chemical barrier and can propagate inflammatory and immune responses, especially when damaged.^3^, ^4^ Multiple studies have shown that cigarette smoke exposure induces significant transcriptional changes throughout the respiratory tract and in blood cells and that genetic profiles are associated with the biological response upon smoking.^5^, ^6^, ^7^, ^8^ Various single nucleotide polymorphisms (SNP) associate with the biological response towards smoking or altered gene expression.^5^, ^9^, ^10^ Of note, not all SNP alter gene expression directly, but some can also influence gene expression in response to a stimulus, which are the inducible expression quantitative trait loci (eQTL).

The formyl peptide receptor (FPR) family consists of three members, all of which are G protein‐coupled cell surface receptors, which can be activated by both bacterial components and mitochondria‐derived damage‐associated molecular patterns (DAMP).^11^ While little is known about the role of FPR2 in COPD, it has been shown that FPR1 downregulation provides protection against emphysema in a mouse model.^12^ FPR1 is expressed on leucocytes and structural cells, including epithelial cells.^13^ Further studies are needed elucidating how FPR expression is regulated and what the effects of smoke are on FPR expression and functioning, in order to unravel the role of FPR in COPD pathophysiology.

Here, we studied whether SNP associate with cigarette smoke‐induced changes in *FPR* family member gene expression in lung epithelial cells and may lead to functional effects in the pathophysiology of COPD. To this end, we performed a candidate‐based inducible eQTL study, investigating the response of respiratory epithelial cells to cigarette smoke. Here, we correlated genetic and transcriptomic data in lung epithelial cells from bronchial brushings of social smokers, which were collected 24 h after smoking three cigarettes within 1 h and after 48 h without smoking.^14^ Furthermore, the effect of inducible eQTL on clinical parameters and lung function was studied, as well as the functional effect of FPR1 on repair of the lung epithelial barrier in the presence of cigarette smoke in wild‐type and CRISPR‐Cas9 knockdown alveolar epithelial cell lines. FPR1 may be a novel therapeutic target for cigarette smoke‐related diseases.

## METHODS

### Study population and design

Bronchial brushes were collected from 65 social smokers with healthy lung function (*n* = 55) or mild COPD (*n* = 10) (study participant characteristics are shown in Table 1). Social smoking was defined as occasional smoking and being able to quit for at least 2 days. Bronchial brushes were collected 24 h after smoking three cigarettes within 1 h and 6 weeks later after at least 48 h without smoking.^14^ Gene expression was measured using the Affymetrix GeneChip Hu_Gene 1.0 ST (Waltham, MA, USA) array and genotyping was conducted using the Illumina Human CytoSNP (San Diego, CA, USA). All subjects provided written informed consent and the study protocol was approved by the Medical Ethical Committee of the University Medical Center Groningen, The Netherlands.

**Table 1:** Characteristics of study participants

	Study subject characteristics
Number of samples, *n*	65
Age	40.43 (18–74)
BMI (kg/m^2^)	24.47 (18.2–32.5)
Sex, male/female	49/16
Pack‐years	16.7 (0–60)
Current smokers, *n* (%)	57
Never smokers, *n* (%)	0
FEV_1_ (% predicted)	96.46 (41.1–135.5)
FEV_1_/FVC (% predicted)	0.92 (0.41–1.17)
Reversibility % from baseline	5.94 (−2.76–23.53)
RV (% predicted)	100.17 (50.5–200.2)
RV/TLC (% predicted)	91.45 (48.8–143.5)

All data are expressed as mean and range.

BMI, body mass index; FEV_1_, forced expiratory volume in 1 s; FVC, forced vital capacity; RV, residual volume; TLC, total lung capacity.

### Genome‐wide inducible eQTL analysis

For the identification of inducible eQTL for the FPR family, a linear mixed‐effect model was run using the R package nlme. We corrected for age and gender in our analysis. Here, the interaction between SNP and gene expression of *FPR1–3* was assessed. For each gene, all SNP located within 1 000 000 base pairs flanking the start and end of the gene were included in the analysis. A dominant model was used for the genotype to increase the group size and to decrease the effect of outliers due to low allele frequency. Not all patients had matched samples due lack of a follow‐up bronchoscopy or RNA of insufficient quality. The following formula was used:

$$\text{Expression}-\text{genotype}\times \text{exposure}+\text{genotype}+\text{exposure}+\mathrm{sex}+\mathrm{age}\;\left(1|\text{patient}\;\mathrm{ID}\right)$$

Inducible eQTL were correlated to the patient characteristics and lung function parameters shown in Table 2.

**Table 2:** The effect of rs3212855 on lung function and blood markers upon smoking

	rs3212855 AA	rs3212855 AC/CC
*n*	55	10
Sex (% male)	76.8	81.8
Age	40 ± 2.4	50 ± 6.4
BMI	24 ± 0.4	26 ± 0.9
% Current smokers	91.1	72.7
Mean no. of cigarettes per day	9.8 ± 1.0	8.1 ± 2.1
Pack‐years	18 ± 2.3	19.8 ± 4.6
FVC (% predicted)	110.2 ± 1.8	103.1 ± 3.5
FEV_1_ (% predicted)***	103.9 ± 2.5	80.9 ± 6.2
FEV_1_/FVC**	0.95 ± 0.02	0.79 ± 0.06
Blood leucocytes	6.5 ± 0.2	7.0 ± 0.5
Blood neutrophils	3.4 ± 0.2	3.7 ± 0.3
Blood eosinophils	0.19 ± 0.02	0.24 ± 0.01
Blood lymphocytes	2.2 ± 0.09	2.2 ± 0.14
Blood CRP*	1.9 ± 0.37	3.0 ± 0.6

Blood markers were measured 24 h after smoking three cigarettes within 1 h. All data are expressed as mean ± SEM. Statistical significance was tested using a Wilcoxon signed‐rank test with Bonferroni correction.

* *P* < 0.05; **P < 0.01; ***P < 0.001.

BMI, body mass index; CRP, C‐reactive protein; FEV_1_, forced expiratory volume in 1 s; FEV_1_/FVC, FEV_1_ divided by the FVC; FVC, forced vital capacity; *n*, number of subjects; rs3212855 AA, individuals homozygous for the major allele of the SNP rs3212855; rs3212855 AC/CC, individuals homozygous for the minor allele of rs3212855 as well as heterozygous individuals; SNP, single nucleotide polymorphism.

### CRISPR‐Cas9‐generated FPR1 knockout A549 cells

Guide RNA (gRNA) was designed using the online webtool Benchling (version 2018, San Fransisco, CA, USA). A gRNA pair was designed for FPR1, which targets a common exon present in both of the known splice variants (Ensembl) and contains the protospacer adjacent motif (PAM) sequence NGG. Furthermore, the gRNA pair was chosen based on the high on‐target cleavage score. The Px458 CRISPR‐Cas9 plasmid was used. CRISPR constructs were amplified using the *Escherichia coli* DH5α strain (NEB 5‐alpha competent *E. coli*) (New England BioLabs, Ipswich, MA, USA) for bacterial transformation. After positive selection and amplification the CRISPR‐Cas9, plasmids were isolated and purified with QIAGEN Plasmid Mini Kit Cat. No. 12123 (QIAGEN, Venlo, The Netherlands) from the bacterial culture according to manufacturer's protocol. The px458 CRISPR‐Cas9 constructs were transfected into the human lung adenocarcinoma cell line A549 (ATCC, Manassas, VA, USA) using Lipofectamine 3000 Transfection Reagent (ThermoFisher‐Scientific, Waltham, MA, USA) according to the manufacturer's protocol. To create a FPR1 knockout cell line, cells were single cell sorted based on green fluorescent protein positivity (SH800S Cell Sorter; Sony Biotechnology, Weybridge, UK). Cells were upscaled and validated using sequencing and western blot. Sequencing data were obtained from BaseClear and FPR1 knockout was assessed with the TIDE Web tool and Benchling. Western blot validation was performed as previously described using an antibody against FPR1 (ab113531; Abcam, Cambridge, UK).^15^ Densitometry of both the FPR1 and beta‐actin, as loading control, bands was determined using ImageJ. FPR1 bands were corrected for total protein levels.

### Cell culture and experimentation

Both the FPR1 knockout and the wild‐type A549 cell line were derived from a sorted single cell. Cells were cultured in RPMI‐1640 growth medium (BioWhittaker, Verviers, Belgium) supplemented with 10% heat‐inactivated foetal calf serum (BioWhittaker) and 100 U/mL penicillin, 100 μL/mL streptomycin (Penstrep; BioWhittaker). Cells were grown in 25‐cm^2^ plastic culture flasks (Costar, Cambridge, MA, USA) at 37°C in an atmosphere of 5% CO_2_ until 90% confluency was reached and then the cells were passaged. Cigarette smoke extract (CSE) was prepared as described previously.^16^, ^17^, ^18^ In short, two filterless 3R4F research cigarettes (Tobacco Research & Development Center, Lexington, KY, USA) were bubbled through 25 mL of RPMI‐1640 medium using a high‐flow peristaltic pump (Watson Marlow 603S, Rotterdam, The Netherlands). The obtained solution was regarded as 100% CSE and was diluted with RPMI‐1640 to obtain a 30% CSE solution. The extract was prepared freshly for each experiment and used within 30 min. Prior to CSE exposure and subsequent wounding by electroporation (30 s, 4500 μA), cells were serum‐starved for 24 h. Epithelial barrier function and repair upon wounding were assessed real‐time using the Electric Cell‐substrate Impedance Sensing (ECIS) Zθ Theta system (Applied Biophysics, Troy, NY, USA) with associated software. Per well, 50 000 cells were seeded into ECIS 8W10E+ arrays (Applied Biophysics) and resistance was monitored at multiple frequencies.^19^

### Statistical analysis

Statistical significance between differential gene expression of the minor and major allele groups was assessed using the Wilcoxon signed‐rank test and differences between conditions of the ECIS measurements over time were assessed using a two‐way analysis of variance (ANOVA) test. *P* < 0.05 was considered as statistically significant.

## RESULTS

### Cigarette smoke‐induced eQTL analysis identifies novel inducible eQTL

**Figure 1:**
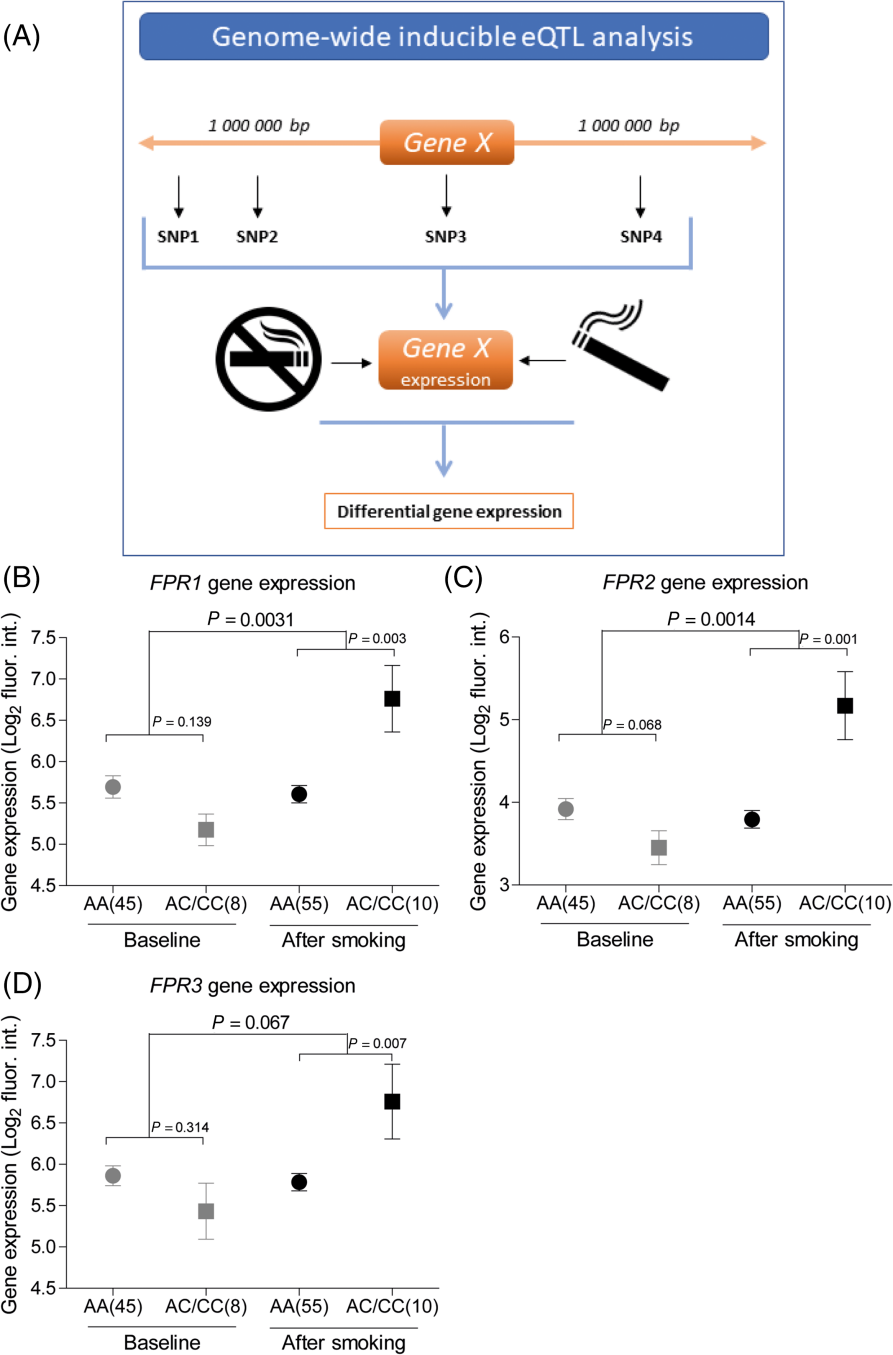
(A) Schematic overview of the experimental set‐up. Inducible expression quantitative trait loci (eQTL) microarray gene expression plots of (B) formyl peptide receptor (FPR) 1, (C) FPR2 and (D) FPR3, which are affected by the interaction of rs3212855 and smoke exposure. The difference in FPR gene expression between the CC and CA groups is analysed for baseline and after smoking. The figure shows the average ± mean for all groups. Significance is tested using a linear mixed‐effect model; *P*‐values are depicted above the graphs and *P* < 0.05 is considered statistically significant. The small *P*‐values depict the analysis between baseline and after smoking, while the large *P*‐values depict the analysis of the expression difference between baseline and after smoking between the AA and AC/CC groups.

To identify SNP that influence FPR gene expression upon smoking, we performed a *cis*‐inducible eQTL study using gene expression of FPR family members and genotyping data in bronchial brushes from 65 social smokers (Table 1). Samples were collected 24 h after smoking three cigarettes within 1 h and 6 weeks later after at least 48 h without smoking.^14^ Smoking‐induced *FPR1–3 cis*‐eQTL were identified by investigating the effect of SNP, located within a 1 000 000 base pair region around a gene, on the difference of its expression between the smoking and non‐smoking samples (Fig. 1A). Here, one eQTL (rs3212855) was identified with a significant interaction of smoking and the effect of SNP genotype on gene expression (FDR < 0.05) and a gene expression fold change of more than 2. rs3212855 strongly associated with smoke‐induced increased expression of *FPR1*, *FPR2* and *FPR3* upon smoking (Fig. 1B–D). rs3212855 Has an allele frequency of 7.6% and is located on chromosome 19, 2 kb upstream of the gene encoding Kalikrein 1 (*KLK1*). However, rs3212855 does not influence the expression of *KLK1* either basally or upon smoking (data not shown).

### rs3212855 Associates with reduced lung function and increased systemic CRP levels

**Figure 2:**
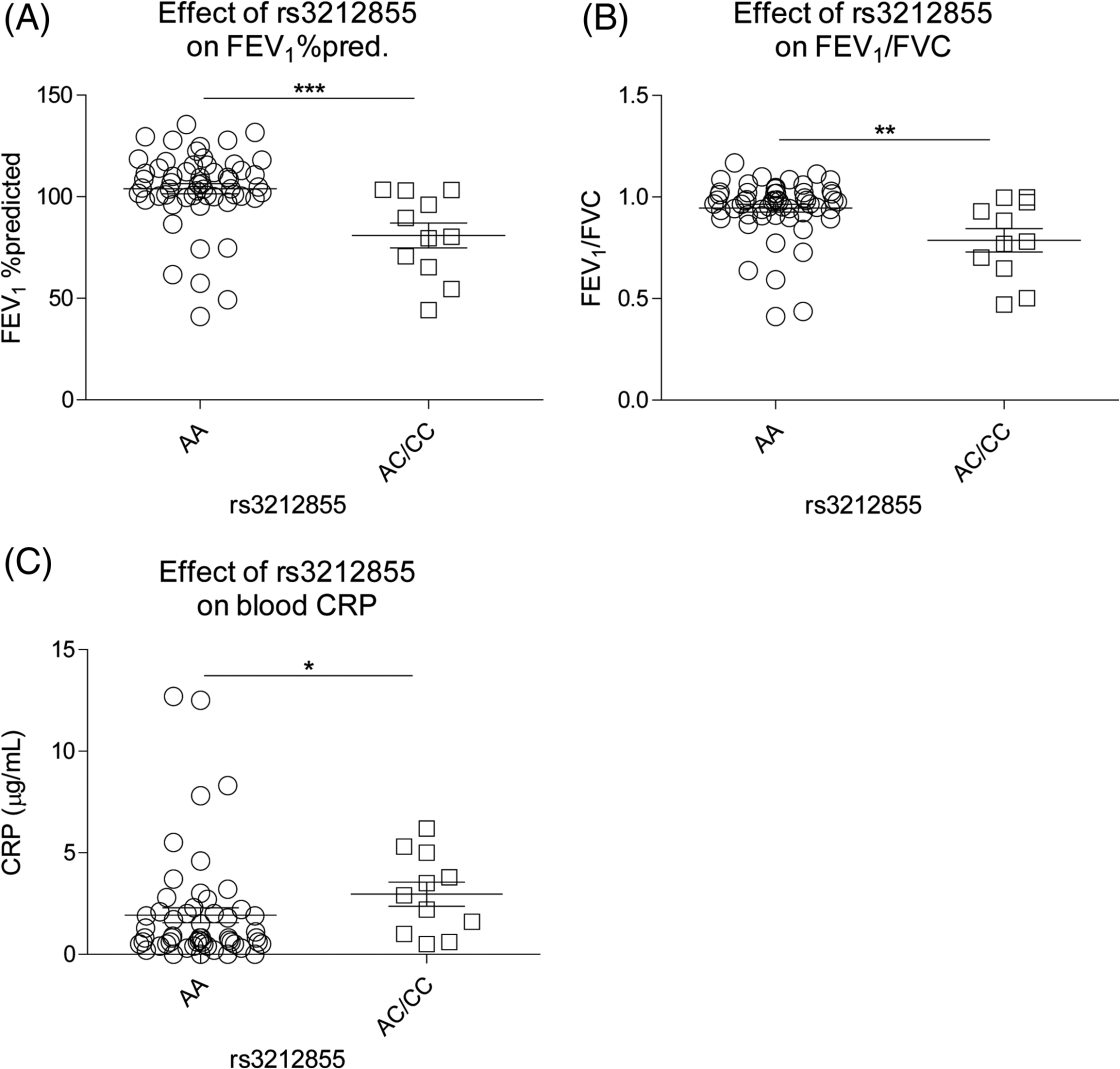
The effect of rs3212855 on lung function and circulating inflammatory markers. (A) The percentage of the predicted forced expiratory volume in 1 s (FEV_1_), (B) FEV_1_ divided by the forced vital capacity and (C) serum C‐reactive protein levels in study participants homozygous for the major allele (*n* = 55) or homozygous for the minor allele together with heterozygous subjects for rs3212855. Statistical significance was tested using a Wilcoxon signed‐rank test with Bonferroni correction. *P < 0.05; **P < 0.01; ***P < 0.001.

Next, it was investigated whether the newly identified inducible eQTL (rs3212855) also associates with clinical characteristics, lung function or blood inflammatory values using the same cohort of subjects as was used to identify the inducible eQTL. Heterozygotes were grouped together with the homozygotes for the minor allele to increase power. Upon investigation of the data obtained after smoking, a significant and strong association between rs3212855 and the forced expiratory volume in 1 s (FEV_1_) was identified (Table 2). Interestingly, rs3212855 did not affect forced vital capacity (FVC), yet the FEV_1_/FVC ratio was significantly associated with the SNP. Furthermore, to investigate the association of rs3212855 with inflammation, blood cell counts and C‐reactive protein (CRP), the mostly used systemic inflammation marker, were measured (Fig. 2). This analysis identified a significant association of rs3212855 with higher CRP levels in the minor allele group. This association was also observed at baseline (Fig. S1A–C in Supplementary Information). Although the neutrophil and eosinophil counts were higher in the minor allele group, no significant association of rs3212855 with inflammatory cell counts was found. Moreover, a significant correlation was identified between the gene expression levels of FPR1 in bronchial brushings and CRP levels in blood, but not with lung function (Fig. S1D,E in Supplementary Information). Taken together, these data suggest that the cigarette smoke‐induced increase in *FPR* family member gene expression does not affect lung inflammation, but may have detrimental effects on lung function.

### FPR1 deficiency protects cells against the detrimental effects of cigarette smoke on lung epithelial repair

**Figure 3:**
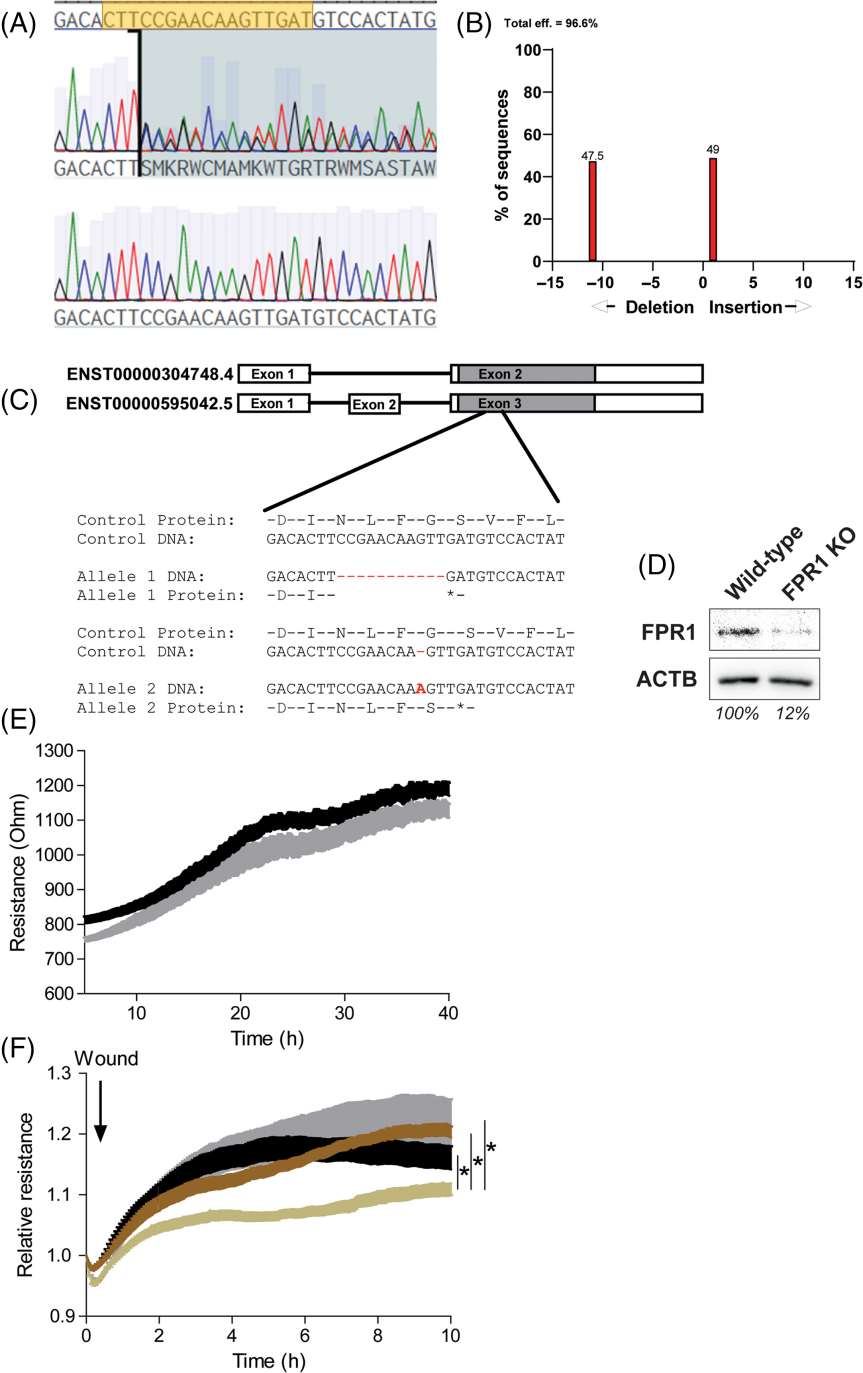
CRISPR‐Cas9‐generated formyl peptide receptor (FPR) 1 knockout (KO) A549 alveolar epithelial cells are protected from the detrimental effects of cigarette smoke extract (CSE) on epithelial wound repair. Using CRISPR‐Cas9, FPR1 KO and wild‐type cells were generated. Both cell lines are derived from a single cell. (A–C) DNA sequencing confirmed the FPR1 KO and showed a 11 base pair deletion on one chromosome and a one base pair insertion on the other chromosome. Both alterations led to the formation of a pre‐mature stop codon. (B) 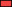, *P* < 0.001. (D) FPR1 downregulation was confirmed using western blot. Representative blot of six independently performed western blots. (E) Transepithelial resistance measured in real‐time using the Electric Cell‐substrate Impedance Sensing (ECIS) system. Both FPR1 KO (black, 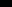) and wild‐type (grey, 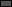) A549 cells formed an epithelial barrier within 2 days. (F) Both FPR1 KO and wild‐type A549 cells were stimulated with 0% or 30% CSE for 1 h before being wounded using electroporation (30 s, 4500 μA). Epithelial repair was followed for 10 h after wounding. Wild‐type A549 cells stimulated with 30% CSE was significantly different from all three other groups, as tested by a two‐way analysis of variance (ANOVA). *P < 0.05. All ECIS experiments were repeated eight times (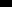, FPR1 KO; 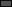, wild‐type; 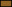, FPR1 KO + 30% CSE; 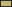, wild‐type + 30% CSE).

Next, we investigated whether FPR1 downregulation provides protection against the cigarette smoke‐induced reduction in lung epithelial repair. We created a CRISPR‐cas9 knockout cell‐line for FPR1 in the human alveolar epithelial cell line A549. The gRNA was designed to target a common exon in both known splice variants of FPR1 (Fig. 3). Using DNA sequencing, we validated the 11 base pair deletion on one chromosome and one base pair insertion on the other chromosome, both producing a pre‐mature stop codon. Lastly, we validated FPR1 downregulation by showing loss of FPR1 protein levels using western blot.

Next, we studied whether FPR1 deficiency impacted alveolar epithelial integrity at baseline and upon wounding in the presence and absence of CSE. Using ECIS, we showed that FPR1 knockout cells did not display abnormalities in barrier integrity during formation of the monolayer (Fig. 3A,B). Next, the repair capacity was assessed by measuring recovery of the monolayer upon wounding by electroporation. This type of wounding results in cell death on the electrode, as indicated by a strong reduction in resistance, after which the surrounding cells fully repopulate the wounded area within 8 h (Fig. 3).^19^ Exposing wild‐type cells to 30% CSE 1 h prior to electroporation strongly reduced the repair capacity of A549 cells (Fig. 3). Interestingly, the FPR1 knockout A549 cells were protected from the negative effects of CSE exposure on the repair response (Fig. 3C,D). Thus, these data indicate that the knockout of FPR1 provides protection against the detrimental effects of cigarette smoke on the repair of alveolar epithelial cells.

## DISCUSSION

This is the first study to identify SNP that associate with cigarette smoke‐induced changes in *FPR* gene expression. Using a novel approach, investigating associations between the genomic SNP profile and the differential gene expression at baseline and after smoking, we identified that FPR are affected by smoke and are thus potentially involved in the pathophysiology of COPD. The most significant hit was rs3212855, which was associated with higher expression of *FPR1*, *FPR2* and *FPR3* upon smoking and lower lung function. Furthermore, knockdown of FPR1 in alveolar epithelial cells provides protection against the cigarette smoke‐induced reduction in their repair responses.

rs3212855 Strongly increases the expression of *FPR1*, *FPR2* and *FPR3* located 921, 928 and 971 kb upstream of rs3212855, respectively. rs3212855 Is not located within a known enhancer or repressor site, leaving the mechanism of action of rs3212855 on FPR expression unknown to date. FPR1, FPR2 and FPR3 all translate into FPR, which are seven‐transmembrane‐spanning G protein‐coupled receptors of the innate immune system that can elicit an immune response upon activation by ‘strangers’, pathogen‐associated molecular patterns PAMP or DAMP.^20^, ^21^ FPR are widely expressed by leucocytes, including neutrophils and monocytes and are also expressed by structural cells, such as epithelial cells and fibroblasts.^22^ FPR can be activated by peptides which bear a formylated methionine, the so‐called N‐formyl‐peptides that are found in bacteria and in mitochondria.^23^ One of the most well‐known formyl peptides, N‐Formylmethionine‐leucyl‐phenylalanine (fMLP), which is readily used to activate granulocytes in vitro, has been found in cigarette smoke.^24^, ^25^ The fMLP in cigarette smoke may activate FPR1/2 receptors, and downstream signalling may be directly involved in impaired epithelial repair responses, explaining the reduced adverse effects of cigarette smoke seen in FPR1 knockout cells. We speculate that impaired lung epithelial repair responses in rs3212855 minor allele‐carrying smokers may contribute to the lung function decline in these individuals. Next to the pro‐inflammatory responses induced by FPR activation, FPR1 and FPR2 are also involved in anti‐inflammatory responses by acting as a receptor for the anti‐inflammatory eicosanoid lipoxin A4.^26^

Little is known about the role of smoke on FPR expression or the role of FPR on smoke‐related diseases like COPD. However, one study showed that the genetic ablation of FPR1 in mice provided protection against cigarette smoke‐induced emphysema and airway inflammation.^12^ These data were further supported by a study showing that the chemical inhibition of FPR reduces chronic cigarette smoke‐induced airway inflammation and alveolar tissue damage.^27^ Furthermore, it was shown that the surface expression of FPR1 on neutrophils isolated from blood was higher in COPD patients compared to healthy non‐smoking controls,^28^ suggesting an aggravated FPR1 pro‐inflammatory response upon smoking in COPD patients. Our study adds to these data that in addition to the expression on neutrophils, the expression of FPR1 on lung epithelial cells may affect repair responses upon smoking and thus impact alveolar damage. Furthermore, we showed that a sub‐population having the minor allele of rs3212855 has reduced lung function as well as reduced smoke‐induced *FPR1* gene expression and that in in vitro studies FPR1 knockout epithelial cells are protected from the detrimental effects of cigarette smoke. Moreover, we showed that FPR1 knockout cells were protected against the detrimental effect of CSE on alveolar epithelial repair responses. These effects can either be direct effect of FPR1 downregulation or can be derived from an indirect effect of secondary pathways affected by FPR1 downregulation.

We did not identify a significant association between FEV_1_% predicted and FPR1 expression, likely because lung function is a complex process dependent of many different factors. However, an association between blood CRP levels and FPR1 expression was shown, likely because CRP levels can be acutely changed by factors like smoking, while changes in lung function are more long‐term chronic effects.

Taken together, we show for the first time that cigarette smoke‐induced alterations in *FPR* gene expression are influenced by specific SNP. In a *cis*‐inducible eQTL study where genetic profiles were associated with cigarette smoke‐induced gene expression alterations, we identified a specific SNP, rs3212855, which regulates the expression of FPR, and is associated with lower lung function. Moreover, we showed that downregulation of FPR1 protects against the detrimental effects of cigarette smoke exposure on epithelial repair. rs3212855 May be used as genetic biomarker for identifying the response towards cigarette smoke and the susceptibility for smoke‐related lung function decline. Furthermore, FPR1 may be a future treatment target for those who are susceptible for the cigarette smoke‐induced decline in lung function.

## Author contributions

Conceptualization: S.D.P., V.R.W., I.H., A.F. Formal analysis: S.D.P., V.R.W., I.E.F., M.B., A.F. Investigation: S.D.P., V.R.W., I.E.F., M.B., A.F. Supervision: S.D.P., I.H., A.F. Resources: N.H.T.t.H., M.v.d.B. Writing—original draft: S.D.P. Writing—review and editing: S.D.P., V.R.W., N.H.T.t.H., M.v.d.B., I.H., A.F. Approval of publication: S.D.P., V.R.W., I.E.F., M.B., N.H.T.t.H., M.v.d.B., I.H., A.F.

## Supporting information


**Figure S1** The effect of rs3212855 on C‐reactive protein (CRP) levels and lung function at baseline and the correlation between FPR1 gene expression with CRP and lung function.

**Visual Abstract** Acute cigarette smoke‐induced eQTL affects formyl peptide receptor expression and lung function.
