# Supplementary material for: Acute cigarette smoke‐induced eQTL affects formyl peptide receptor expression and lung function
Source: Respirology. 2020 Oct 19;26(3):233–40. doi: 10.1111/resp.13960 (PMC7983955; doi:10.1111/resp.13960)
Supplement: Supplementary file 1 — Figure S1 The effect of rs3212855 on C‐reactive protein (CRP) levels and lung function at baseline and the correlation between FPR1 gene expression with CRP and lung function. [file RESP-26-233-s001.docx]

**Supplementary Information**

**Acute cigarette smoke-induced eQTL affects formyl peptide receptor expression and lung function**

Simon D. Pouwels*^1,2,3^, Valerie R. Wiersma*^4^, Immeke E. Fokkema^1^, Marijn Berg^1,3^, Nick H.T. ten Hacken^2^, Maarten van den Berge^2,3^, Irene Heijink**^1,2,3^, Alen Faiz**^5^.

1. Department of Pathology & Medical Biology, University Medical Center Groningen (UMCG), University of Groningen, Groningen, The Netherlands
2. Department of Pulmonology, University Medical Center Groningen (UMCG), University of Groningen, Groningen, The Netherlands
3. Groningen Research Institute for Asthma and COPD (GRIAC), University Medical Center Groningen (UMCG), University of Groningen, Groningen, The Netherlands
4. Department of Hematology, Cancer Research Center Groningen, University Medical Center Groningen (UMCG), University of Groningen, Groningen, The Netherlands
5. Respiratory Bioinformatics and Molecular Biology, University of Technology Sydney, Sydney, New South Wales, Australia

*SDP and VRW contributed equally to this study.

** IH and AF contributed equally to this study.


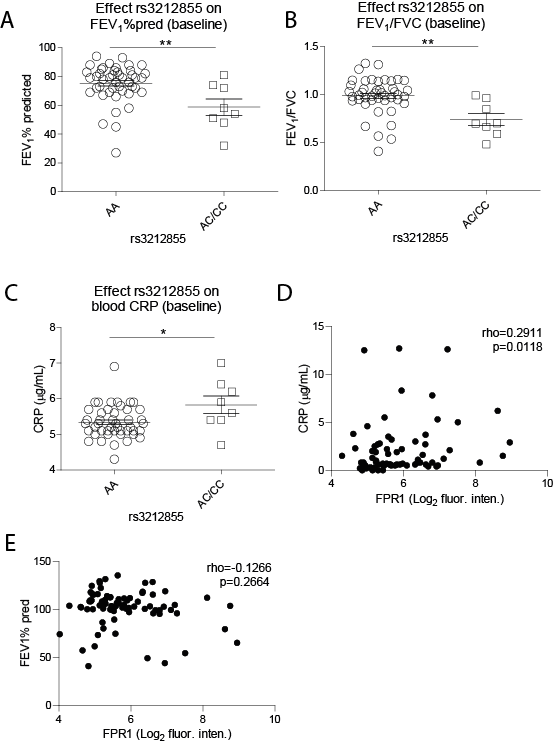


***Figure S1: The effect of rs3212855 on CRP levels and lung function at baseline and the correlation between FPR1 gene expression with CRP and lung function.***

***A)*** *The percentage of the predicted Forced Expiratory Volume in 1 second,* ***B)*** *the FEV1 divided by the Forced Vital Capacity, and* ***C)*** *serum C-Reactive Protein (CRP) levels are shown in study participants homozygous for the major allele (n=55) or homozygous for the minor allele combined with heterozygous subjects, for rs3212855. Lung function and CRP measurements were performed after 48 hours without smoking. Statistical significance was tested using a Mann Whitney U with Bonferroni correction, *=P<0.05, **=P<0.01, ***=P<0.001. Correlation between FPR1 expression in bronchial brushings after smoking and* ***D)*** *C-reactive protein (CRP) or* ***E)*** *the FEV1%pred. Significance was tested using the Spearman's rank-order correlation test, showing the Spearman's correlation coefficient (rho) and the P value, where P<0.05 is considered statistically significant.*
