# Supplementary material for: Acute cigarette smoke‐induced eQTL affects formyl peptide receptor expression and lung function
Source: Respirology. 2020 Oct 19;26(3):233–40. doi: 10.1111/resp.13960 (PMC7983955; doi:10.1111/resp.13960)
Supplement: Supplementary file 2 — Visual Abstract Acute cigarette smoke‐induced eQTL affects formyl peptide receptor expression and lung function. [file RESP-26-233-s002.pptx]

## Slide 1
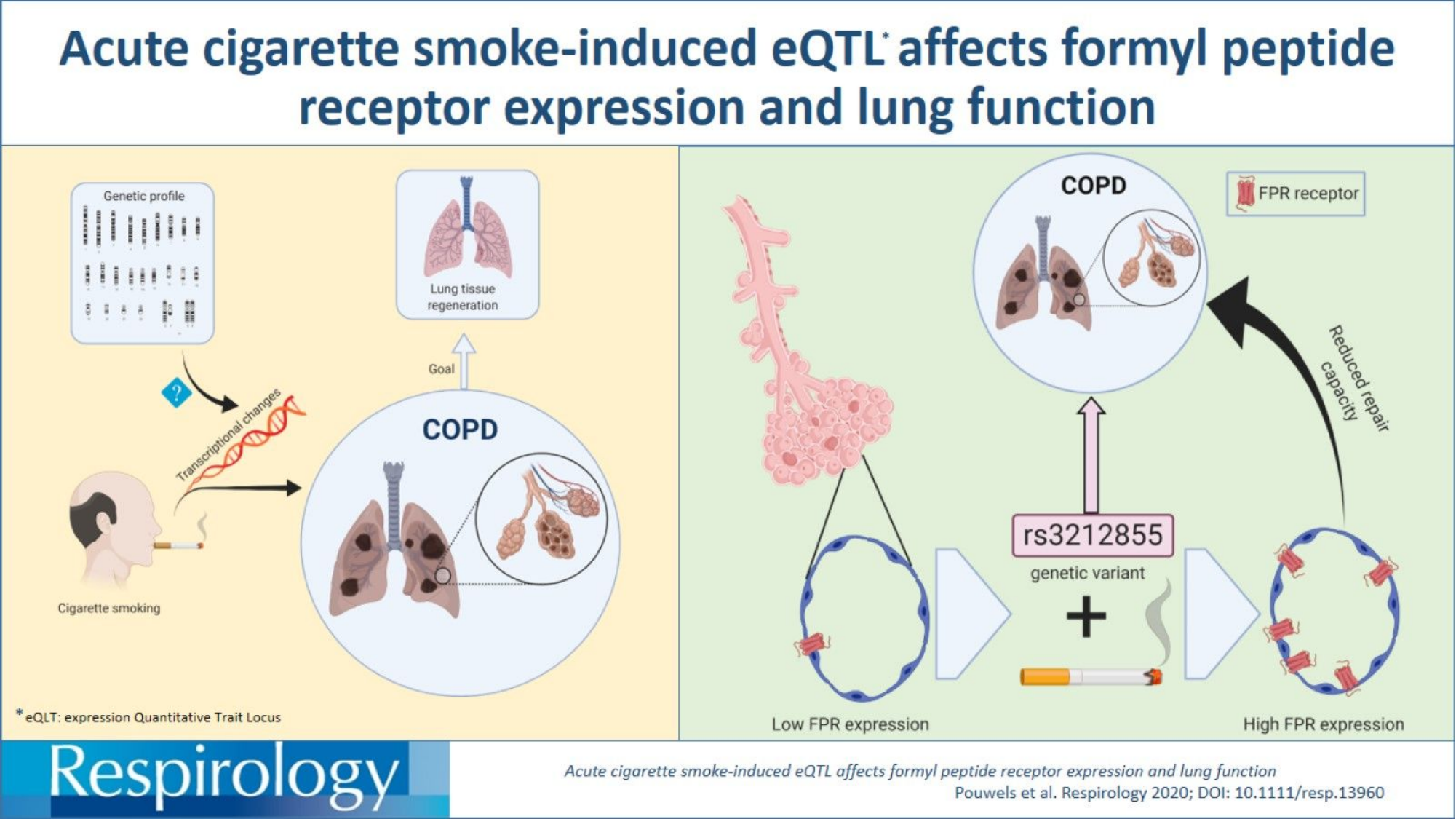

# Acute cigarette smoke-induced eQTL affects formyl peptide receptor expression and lung function
